# Effects of Green Tea Compound Epigallocatechin-3-Gallate against *Stenotrophomonas maltophilia* Infection and Biofilm

**DOI:** 10.1371/journal.pone.0092876

**Published:** 2014-04-01

**Authors:** Pedrina G. Vidigal, Mathias Müsken, Katrin A. Becker, Susanne Häussler, Jost Wingender, Eike Steinmann, Jan Kehrmann, Erich Gulbins, Jan Buer, Peter Michael Rath, Jörg Steinmann

**Affiliations:** 1 Institute of Medical Microbiology, University Hospital Essen, University of Duisburg-Essen, Essen, Germany; 2 Institute for Molecular Bacteriology, TWINCORE, Center for Experimental and Clinical Infection Research, Hannover, Germany; 3 Department of Molecular Bacteriology, Helmholtz Center for Infection Research, Braunschweig, Germany; 4 Institute of Molecular Biology, University of Duisburg-Essen, Essen, Germany; 5 Biofilm Center, Department of Aquatic Microbiology, Faculty of Chemistry, University of Duisburg-Essen, Essen, Germany; 6 Division of Experimental Virology, TWINCORE, Center for Experimental and Clinical Infection Research, Hannover, Germany; Charite Universitätsmedizin Berlin, Germany

## Abstract

We investigated the *in vitro* and *in vivo* activities of epigallocatechin-3-gallate (EGCg), a green tea component, against *Stenotrophomonas maltophilia* (Sm) isolates from cystic fibrosis (CF) patients. *In vitro* effects of EGCg and the antibiotic colistin (COL) on growth inhibition, survival, and also against young and mature biofilms of *S. maltophilia* were determined. Qualitative and quantitative changes on the biofilms were assessed by confocal laser scanning microscopy (CLSM). Further, *in vivo* effects of nebulized EGCg in C57BL/6 and *Cftr* mutant mice during acute Sm lung infection were evaluated. Subinhibitory concentrations of EGCg significantly reduced not only biofilm formation, but also the quantity of viable cells in young and mature biofilms. CLSM showed that EGCg-exposed biofilms exhibited either a change in total biofilm biovolume or an increase of the fraction of dead cells contained within the biofilm in a dose depended manner. Sm infected wild-type and *Cftr* mutant mice treated with 1,024 mg/L EGCg by inhalation exhibited significantly lower bacterial counts than those undergoing no treatment or treated with COL. EGCg displayed promising inhibitory and anti-biofilm properties against CF Sm isolates *in vitro* and significantly reduced Sm bacterial counts in an acute infection model with wild type and CF mice. This natural compound may represent a novel therapeutic agent against Sm infection in CF.

## Introduction

Bacterial pathogens are progressively reported as an important cause of high morbidity and mortality rates among patients with cystic fibrosis (CF) [1]. The success of available conventional antibiotic therapies in eradicating bacterial infections in CF patients is limited because the resistance exhibited by these microorganisms is increasing [2]. One plausible explanation for this increasing resistance is the fact that bacteria can form biofilms, a type of microbial community enveloped by extracellular polymeric substances, in which they are subjected to selective mutational pressures probably induced by repeated antibacterial treatments over the long term [1], [3]–[5]. In addition, these biofilms usually reduce the penetration of antibiotics or induce the expression of more complex biofilm-specific resistance mechanisms [6]–[7]. Therefore, there is an increased need for novel drugs that can overcome this obstacle [8]–[10].

Although studies have shown associations between infections caused by *S. maltophilia* with increased risk of developing pulmonary exacerbation, lung transplantation and death [1]–[12], it is still unclear whether this pathogen is simply a marker of the disease's severity or if it is causally linked to the CF disease progression. Various CF centers worldwide have reported an increased prevalence of Sm [1]. It is a multi-drug resistant, opportunistic pathogen that often causes nosocomial infections (e.g. pneumonia) [13]. Furthermore, this Gram-negative rod is recognized by its ability to form biofilms on abiotic surfaces including glass and plastics like polystyrene, as well as on host tissues such as bronchial epithelial cells [13]–[14].

Epigallocatechin-3-gallate (EGCg) is the most abundant polyphenol found in green tea (*Camellia sinesis*). Notably, *in vitro* studies have shown that EGCg is an effective antimicrobial compound against a variety of Gram-positive and Gram-negative bacterial, as well as fungal pathogens [15]–[19]. Further investigations have shown that EGCg indeed has antimicrobial effects against *Pseudomonas aeruginosa* and *Staphylococcus aureus*, two of the most relevant pathogens in patients with CF [20]–[21]. Colistin (COL) is a polymyxin antibiotic effective against Gram-negative bacteria. Because of the low level of reported resistance, this antibiotic is considered the frontline treatment for infections caused by intermittent colonisation of Gram-negative rods in CF [22]. The purpose of this study was to investigate the antimicrobial activity of EGCg against CF Sm isolates and acute pulmonary Sm infection induced in wild type and *Cftr* mutant mice. Further, we determine EGCg effects on biofilms in comparison to that of COL.

## Materials and Methods

### Antimicrobial agents

EGCg and COL were obtained from Sigma (Sigma-Aldrich, St Louis, MO, USA). Stock solutions (1,024 mg/L) of EGCg were freshly prepared and diluted in Mueller-Hinton broth (MHB; Oxoid, Wesel, Germany) containing 1% (v/v) dimethyl sulfoxide (DMSO). COL stock solutions (2,048 mg/L) were also dissolved and diluted in MHB including 1% DMSO. COL stock solution was stored at −20°C until use. For *in vivo* experiments, EGCg and COL were either dissolved in PBS (wild type mice experiments) or aqua (*Cftr* mutant mice experiments) at room temperature.

### Bacterial susceptibility testing of EGCg

A collection of 60 different clonal Sm isolates from CF patients were selected for investigation of the *in vitro* activity of EGCg. Sputum samples were collected from the patients as part of standard care. Bacterial cultures are collected as part of our epidemiological surveillance. EGCg susceptibility profiles of the clinical isolates and reference strain (ATCC13637) were determined using broth microdilution guidelines proposed by the Clinical and Laboratory Standards Institute (CLSI) [23] (see also File S1).

### Time-kill assays

The kinetics of the bactericidal effect of EGCg on Sm was investigated against the strain ATCC 13637 (control) and two CF clinical isolates (obtained from an intermittent and a chronically colonized patients, respectively designated as Sm1 and Sm2) in a microtiter plate assay as previously described [17]. The clinical isolates Sm1 and Sm2 were chosen as their MIC and MBC are representative for the collection of strains and they are strong biofilm producers. The reduction of 2,3-bis (2-methoxy-4-nitro-5-sulfophenyl)-2H-tetrazolium-5-carboxanilide inner salt (XTT) was used as a marker of cell viability. The plates were incubated for 0, 2.5, 5, 10, or 24 h at 36°C. XTT reduction was measured colorimetrically at 492 nm (Sunrise Microplate Reader, Tecan, Männerdorf, Switzerland).

### Effects of inhaled EGCg, administrated pre and post pulmonary infection, in wild type and Cftr mutant mice

All animal experiments were approved by the ethical committee of Landesumweltamt Nordrhein-Westfalen (Q1299/12), Germany. We investigated the antimicrobial effects of EGCg in comparison to COL in female C57BL/6 specific-pathogen-free mice purchased from Harlan Laboratories (Roosdorf, Germany) at 6 to 8 weeks of age. C57BL/6 mice were nebulised for 5 min with 1×PBS (n = 13), 128 mg/L COL (n = 12) or 1,024 mg/L EGCg (n = 11). Infection with Sm1 was performed as previously described (see also File S1) [24]. One hour post-infection, nebulisation was performed again as stated above. The clinical aspects of mice were assessed and scored as described previously [25]. Mice were sacrificed by cervical dislocation 4 h post-infection, and lungs were aseptically removed to determine bacterial load. The antimicrobial effects of EGCg were also investigated in female and male *Cftr* mutant mice B6.192P2 (CF/3)-*Cftr* ^TgH (neoim)Hgu^ (abbreviated *Cftr* ^-/-^) at the age of 12 to 14 weeks. *Cftr* mutant mice) were nebulised either with sterile distilled water (n = 10) to prevent any potential mucus clearance or 1,024 mg/L EGCg (n = 10). All mice were maintained in isolated cages to provide a pathogen-free environment at the Central Laboratory Animal Facility of the University Hospital Essen, Essen, Germany.

### Biofilm formation assay

The biofilm assay was performed as previously described [26] with slight modifications (see also File S1). The minimum cut-off point was defined as 3× standard deviation (SD) above the mean OD of control wells (OD_C_), and isolates were classified as follows: no biofilm producer (OD≤OD_C_), weak biofilm producer (OD_C_≤OD≤2×OD_C_), moderate biofilm producer (2×OD_C_≤OD≤4×OD_C_), and strong biofilm producer (4×OD_C_<OD) (23).

### Effect of EGCg on biofilm formation

The effects of EGCg and COL on Sm biofilm formation were determined by the method previously described [18], [26]. ATCC 13637 (control strain) and the clinical isolates (Sm1 and Sm2) were treated with EGCg and COL at 0.25×MIC, 0.5×MIC, or 1×MIC (Table 1). Control wells contained only TSB.

**Table 1:** Susceptibility of Sm isolates to COL and EGCg as determined by the reference microdilution method of the Clinical and Laboratory Standards Institute.

Compounds	ATCC13637		Sm1		Sm2	
	MIC	MBC	MIC	MBC	MIC	MBC
COL (mg/L)	158	256	256	256	256	256
EGCg (mg/L)	8	32	32	128	32	64

### Reduction of cell viability of EGCg on mature biofilm

To evaluate the reduction of cell viability of EGCg, we cultivated Sm biofilms (ATCC13637, Sm1 and Sm2) in a 96-well microtiter plate assay as previously described [27] (see also File S1). The *in vitro* effect of EGCg on the viability of Sm biofilm was plotted as the ratio of viability (cells with active metabolism) in treated samples to viability in untreated samples. The effects of COL on mature biofilms were determined as described for EGCg.

### Confocal laser scanning microscopy of Sm biofilms

Qualitative and quantitative microscopic evaluations of the biofilms were carried out through a combination of the LIVE/DEAD *Bac*Light viability staining and automated confocal laser scanning microscopy (CLSM), as previously described [28]. The 48-h-old biofilms of strains ATCC 16367, Sm1, and Sm2 were visualised after 24 h exposure to EGCg or COL at various concentrations. For this assay, the DNA-binding dyes Syto9 (green) and propidium iodide (PI; red) were used. This two-colour kit differentially stains living (green) and membrane-compromised/dead (red) bacteria according to differences in membrane permeability. Biofilm susceptibility was determined on the basis of the fractions of red (including co-localized) and green biovolume [μm^3^] calculated from the image stacks with a customer-designed solution in the software Developer XD (Definiens). The negative controls were biofilms treated with Luria Bertani (LB) medium supplemented with 1% (v/v) DMSO, and the positive controls (killing control) were treated with formalin at final concentration of 1% (v/v) formalin. Visualization of biofilm sections was performed with the software IMARIS (Bitplane). Data are expressed as means of two independent experiments. Experiments were carried out in duplicates.

### Statistical analysis

All assays were performed in triplicate, and the results are shown as means ± SDs. A one-way analysis of variance (ANOVA), followed by the Dunnett test, was used to detect differences in activity against biofilm formation (spectrophotometry OD readings) and biovolume/bioviability of young and mature biofilms between isolates exposed to EGCg or COL for 24 h. The Mann-Whitney U test was used to address the differences among bacterial counts in mice nebulized with EGCg, COL, PBS and aqua. Significance was set at *P*<0.05.

## Results

**Figure 1:**
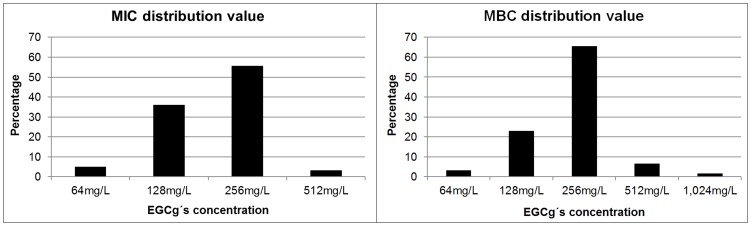
Distribution of minimum inhibitory concentration and minimum bactericidal concentration values determined by microdilution broth assay. Values are expressed as percentage of data obtained from 60 cystic fibrosis (CF) Sm isolates against EGCg. MIC =  minimum inhibitory concentration; MBC =  minimum bactericidal concentration.

**Figure 2:**
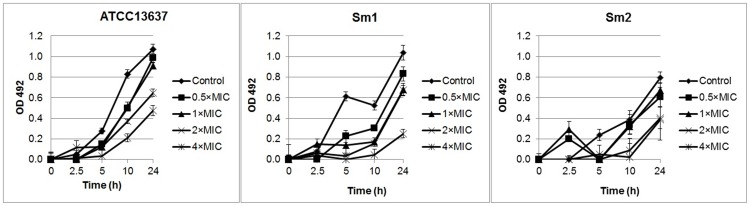
Kinetics of the killing effect of EGCg on Sm ATCC 13637 and two clinical isolates. The concentrations of EGCg ranged from 0.5×MIC to 4×MIC. Bacterial viability over a 24 h period was determined by measurement of optical density (OD) at 492 nm by XTT conversion. Control samples consisted of bacterial cells grown in tryptic soy broth (TSB) in the absence of EGCg. Experiments were designed in three independent sets performed in octuplicate, and the results are expressed as means ± standard deviation (SD).

The antibacterial activity of EGCg was tested *in vitro* against 60 CF Sm isolates. EGCg was active against all isolates; MIC values ranged from 64 to 512 mg/L, and MBC values ranged from 64 to 1024 mg/L (Figure 1). Both the MIC and the MBC at which 50% and 90% of isolates were inhibited and killed were 256 mg/L. The MICs and MBCs values of the three test strains (Table 1). The time-killing curve analysis showed that EGCg was partially bactericidal against reference strain ATCC 13637, Sm1 and Sm2 at 2×MIC and 4×MIC over a 24-h incubation period (Figure 2).

**Figure 3:**
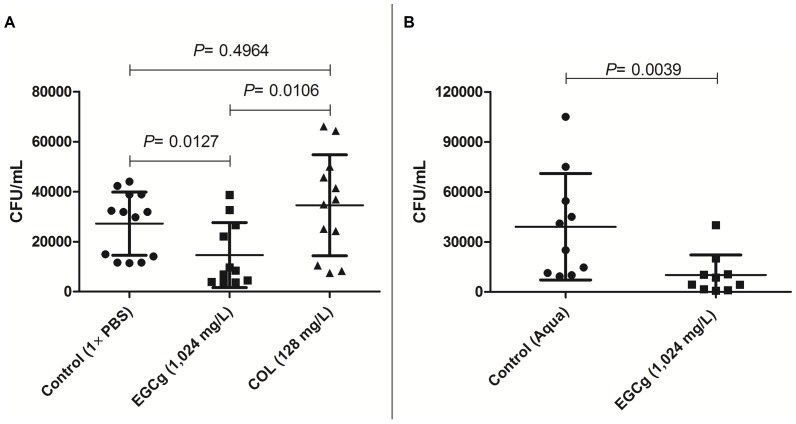
Bacterial load after intratracheal instillation of Sm in C57BL/6 and *Cftr* mutant mice. A) Bacterial count in the lungs of C57BL/6 mice after infection with Sm1 nebulized (2 h before infection and 1 h post-infection) with 1×PBS (n = 13), COL (n = 12) and EGCg (n = 11). Mice nebulized with EGCg exhibited significantly lower bacterial count (P = 0.0127) in comparison to non-treated group (1×PBS). Shown are mean ± SD and the distribution of the values. B) Bacterial count in the lungs of *Cftr* mutant mice after infection with Sm1 nebulized (2 h before infection and 1 h post-infection) with aqua (n = 10) and EGCg (n = 10). Mice nebulized with EGCg exhibited significantly lower bacterial count (P = 0.0039) in comparison to the non-treated group (aqua). Displayed are means ±SD and individual values. Aqua  =  sterile distilled water.

We further evaluated if EGCg could be useful *in vivo* as a novel natural compound for treatment (prophylactic and therapeutic) against acute pulmonary infection caused by Sm1 strain in wild type mice in comparison to COL (Figure 3A). It is important to mention that none of the infections resulted in death, since we performed euthanasia before the overall fitness of the mice were considered severe. We demonstrated that wild type (C57BL/6) mice nebulised with EGCg exhibited significantly lower CFU/mL (*P* = 0.0127) in the lungs compared to untreated (but infected controls) and COL-treated (P = 0.0106). Infected mice receiving COL did not show significantly lower CFU's in the lungs in comparison to controls (*P* = 0.4964). Interestingly, experiments with *C. elegans*, considered a versatile platform for drug discovery, have shown similar results when carried out under the same conditions (see also File S1 and Figure S2). In addition, uninfected nematodes 48 h exposed to different concentrations of EGCg did not exhibit significant lethal effects (see also File S1 and Figure S1). This data encouraged us to examine whether EGCg also protects against pulmonary Sm infections in *Cftr* mutant mice. The results reveal that bacterial counts in the lungs of *Cftr* mutant mice nebulized with EGCg were significantly lower than in those nebulized with sterile distilled water (Figure 3B).

Since the main obstacles for the successful eradication of Sm, especially in CF patients, are its multi-drug resistance profile and its biofilm mode of growth, we investigated the ability of the studied isolates to form biofilm. Most CF Sm isolates (90.0% out of 60 isolates) adhered to and formed biofilm on the polystyrene plates; only 10.0% were considered not to produce biofilm. The isolates were classified as weak (11.7%), moderate (15.0%), or strong (63.3%) biofilm producers. Strains ATCC 13637, Sm1 and Sm2 were considered strong biofilm producers.

**Figure 4:**
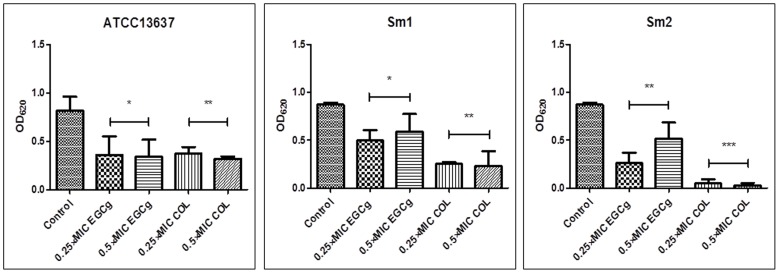
Effects of COL and EGCg against Sm biofilm formation. Reference strain ATCC13637 and two clinical isolates (Sm1, and Sm2) were used. Biofilms were stained with crystal violet and their biomasses were determined by optical density (OD) measurement at 620 nm. Compared to untreated control cells, samples exposed to EGCg and COL exhibited a significant reduction in the number of Sm sessile cells of ATCC13637 and Sm1. Results are expressed as average OD ± standard deviation (SD). Experiments were performed in triplicate. *P<0.05; **P<0.01; ***P<0.0001.

The activity of EGCg and COL was then examined at 0.25×MIC and 0.5×MIC to ascertain if these compounds have an inhibitory effect on biofilm development from the three tested strains in the growth medium TSB (Figure 4). In comparison to the positive (untreated controls), ATCC13637, Sm1 and Sm2 biofilms treated with EGCg and COL (0.25×MIC and 0.5×MIC) displayed biofilm growth reduction in comparison to the positive (untreated) controls. ANOVA results showed significant reduction of biomass in all isolates. These results indicate that EGCg and COL exert anti-biofilm effects at subinhibitory concentrations.

**Figure 5:**
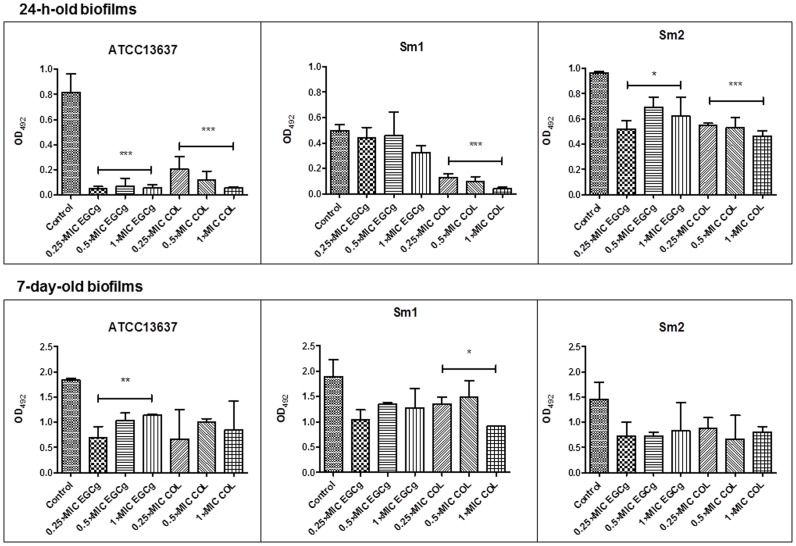
Effects of COL and EGCg on 24-h- and 7-day-old established biofilms of Sm. Reference strain ATCC13637 and clinical isolates (Sm1 and Sm2) had their biofilm metabolic activity defined by XTT viability assay. OD measurement was determined at 492 nm, and results are expressed as average OD ± standard deviation (SD). Experiments were performed in triplicate. *P<0.05; **P<0.01; ***P<0.0001.

Since biofilms play an important role in bacterial persistence, we further assessed the *in vitro* dynamics of mature biofilms exposed to EGCg or COL (Figure 5). After 24 h exposure to EGCg, both 24-h-old and 7-day-old biofilms from ATCC 13637, Sm1, Sm2 showed a mean viability decrease in comparison to untreated biofilms. COL also displayed a reduction of viable cells in the 24-h-old biofilms and 7-day-old biofilms produced by the same strains with relation to the same untreated controls. EGCg had significantly reduced the metabolic activity of young biofilm cells produced by ATCC 13637 and Sm2 samples. Only mature biofilm cells of ATCC 13637 were significantly reduced when treated with EGCg. COL exhibited significant inhibitory effects against young biofilms cells of all tested samples. Only mature biofilm cells of Sm1 were significantly decreased by COL. Additionally, the relative effects of EGCg and COL on the viability of mature biofilms were found not to be dose-dependent.

**Figure 6:**
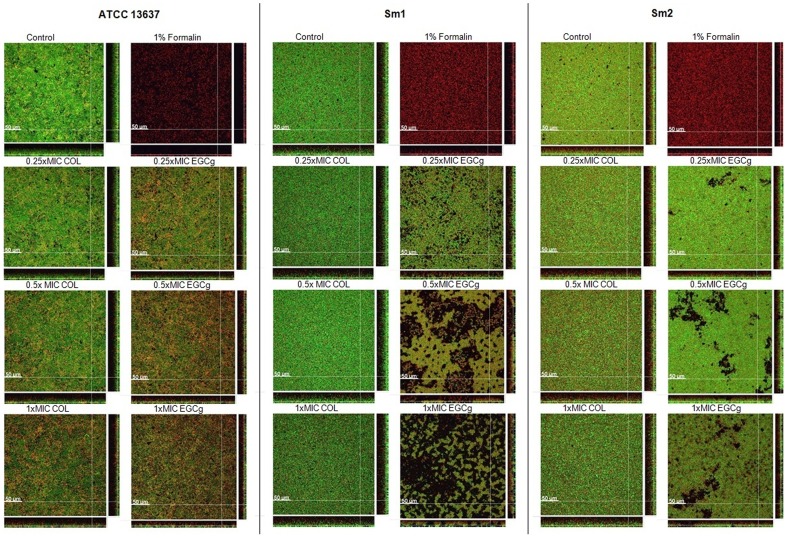
Optical sections of 48-h-old Sm biofilms (reference strain ATCC13637 and clinical isolates: Sm1 and Sm2) treated with EGCg and COL at 0.25×MIC, 0.5×MIC, 1×MIC. Biofilms were treated with formalin as killing control. Live bacteria are stained in green (Syto9), dead bacteria in red (propidium iodide [PI]) or yellow (overlapping regions). Experiments were performed in duplicates (image data: 1024 ×1024 pixel with a pixel-size of 0.284 μm; z-step-size: 2 μm). Length of size bar: 50 μm.

To further substantiate the effects of EGCg and COL on the morphology and viability we performed CLSM of 48-h-old biofilms produced by three strains of Sm. Representative biofilm sections of the acquired image stacks are shown in Figure 6. As visible in the images, biofilms produced by isolates Sm1 and Sm2 showed significant differences in biofilm morphology with increasing concentrations of EGCg in contrast to COL treated biofilms (Figure 6). The ATCC13637 strain was neither structurally altered by EGCg nor by COL; however, the proportion of membrane-compromised/dead cells increased in the presence of higher concentrations of the above mentioned substances. Interestingly, the proportion of membrane-compromised/dead cells of Sm2 was not changed although the structure was altered. In addition to the relative values, the quantitative data illustrates the reduction of total biovolume at highest concentrations (1xMIC) for all samples, except for COL treated ATCC 13637.

## Discussion

In this study we demonstrated the inhibitory activities of EGCg (the main component of green tea) on Sm *in vitro* and compared its antimicrobial effects on young and mature biofilms to those of COL. Despite the bactericidal effects of EGCg on CF Sm isolates at 2×MIC and 4×MIC, subinhibitory concentrations of EGCg exhibited the ability to prevent biofilm formation *in vitro* and to impair structure and viability of pre-established biofilms in a strain-dependent manner. Another approach to verify if EGCg could have any *in vivo* potential antimicrobial effect, Sm infected wild-type and *Cftr* mutant mice treated with 4×MIC EGCg administered to the airways had significant lower bacterial counts than those non-treated or treated with COL.

First, we examined the antibacterial activity of EGCg. Previous studies have demonstrated that EGCg exerts antimicrobial activity against a variety of organisms, including *S. maltophilia* [15]–[18]. Our MIC data (MIC_50/90_ = 256 mg/L) and the time-kill results obtained from CF *S. maltophilia* isolates were similar to those obtained by Gordon and Wareham [17] with a cohort of 40 clinical isolates from non-CF patients.

To test the antibacterial efficacy of EGCg *in vivo*, we performed intratracheal instillation infection in wild type and CF mice. Our results showed that EGCg significantly reduced the bacterial counts in the lungs (Figure 3), suggesting a partial bactericidal effect and possible suppression of bacterial dissemination. Administration of COL as sole antimicrobial agent was not as effective as EGCg, a fact that has been previously reported for infection caused by *P. aeruginosa* in a different *in vivo* model [29]. These data show for the first time that nebulized EGCg seems to be a promising therapy against pulmonary infection caused by Sm in CF patients.

MIC and MBC *per se*, which are conventionally derived from assay using planktonic cells, do not provide sufficient information about the efficacy of antimicrobial agents against bacteria that live in biofilms. Biofilm formation is a survival strategy for bacteria, since biofilm-specific traits such as slow growth rate and low metabolic activity as well as the production of a protective matrix of extracellular polymeric substances contribute to pathogen resistance and are responsible for poor host response [30].

In our study, a high percentage (90.0%) of the CF Sm isolates produced biofilm on polystyrene surfaces. These findings are in line with the results obtained by Di Bonaventura and co-workers, who found that the ability of Sm strains to form biofilm is influenced by environmental conditions (temperature, oxygen availability, and pH) [31]. Because the biofilm mode of growth is an effective defence mechanism in the CF lung, we focused on assessing the impact of COL and EGCg on biofilm formation and on various maturation stages of the biofilm.

We verified that COL and EGCg at subinhibitory concentrations noticeably decreased biofilm formation and cell viability as well. The strongest effect was observed with COL and EGCg at 0.5×MIC. It has been shown that quinolones also exhibit anti-biofilm properties against Sm at sub-MIC concentrations [32]–[33]. Ciprofloxacin, grepafloxacin, and norfloxacin significantly reduced both the biomass and the viability of Sm biofilm at 0.25×MIC [32]. In another study, the same research group also found that low subinhibitory concentrations of moxifloxacin induced a significant decrease in adhesion and biofilm formation of two CF Sm isolates [33].

We examined for the first time whether bactericidal and/or subinhibitory concentrations of COL and EGCg were capable of damaging young and mature biofilms. COL and EGCg reduced 24-h- and 7-day-old biofilms biovolume at various concentrations *in vitro*. Interestingly, when biofilm susceptibility testing was performed and estimated by CLSM, stronger effects were observed after EGCg treatment both with regard to morphology and viability. COL only reduced the total biovolume of both clinical isolates which, however, was already observed by crystal violet staining in Figure 4.

Since no anti-biofilm therapies are clearly established, there is a great need to develop effective strategies for preventing or controlling biofilm-associated bacterial infections. Green tea is not only consumed as a beverage but is also considered a millenary method of traditional medicine in most of Asia; interest in its medicinal properties is increasing in the Western world [34]. Currently, several epidemiological studies have demonstrated that green tea is associated with health benefits in patients with cancer, cardiovascular diseases, and neurological diseases, and that it also exerts antimicrobial effects [33], [35]. Sub-MIC concentrations of EGCg also exert anti-biofilm activity against other pathogens, such as *Streptococcus mutans*, S*taphylococcus aureus*, and *Candida albicans* [19], [36]–[37]. Safety studies have shown that EGCg exhibits low toxicity at high concentrations [38]–[39] and also in human normal lung cells [40], suggesting it as a possible candidate for development as a new therapy for respiratory infection in CF patients. One pharmacokinetic obstacle to the therapeutic use of EGCg is its low oral bioavailability; however, nebulisation therapy could be a promising strategy for improving mucociliary clearance and respiratory function among CF patients [41]. For example, a clinical study verified that inhalation of green tea extract solution by disabled elderly patients eradicated Methicillin-resistant *Staphylococcus aureus* from the upper respiratory tract [42].

The precise mechanisms involved in the activity of EGCg against bacterial growth are still poorly defined. For instance, it has been suggested that EGCg's mechanism of action on Sm is associated with its antifolate activity, which will consequently lead to disruption of DNA synthesis [43]. On the other hand, it has been proposed that catechins play a crucial role by damaging bacterial membranes [15], [44]. Recently, atomic force microscopy has shown that the important morphological changes of cell surfaces of Gram-negative bacteria induced by EGCg are highly dependent on the release of hydrogen peroxide (H_2_O_2_) [21]. Normally, biofilms exhibit high level of activity at the surface but low activity or even slow growth or no growth in the center [42]. It is hypothesized that the anti-biofilm activity of EGCg is not dependent on metabolic activity but is associated with its ability to bind and damage bacterial membranes [21].

Although this work shows clearly that EGCg has beneficial effects, future research needs to solve the current limitations. Prophylactic and therapeutic effects of EGCg *in vivo* were only evaluated simultaneously. Thus, further studies should aim to analyse whether EGCg alone could work as either a prophylactic or therapeutic treatment. Due to the scope for this research, we were unable to verify the antimicrobial effects of EGCg on *in vivo* biofilm. The assessment of chronic bacterial infections *in vivo* can differ from data obtained from *in vitro* experiments. *In vivo* experiments can provide a better insight to the microenvironmental circumstances associated with biofilm and the defence mechanisms exhibited by the host [45]. However, such CF complex model of chronic infection is still not well defined. Further studies should focus on developing models in which long-term exposure of EGCg could be examined during Sm chronic infection.

In summary, this study is the first to evaluate the *in vitro* and *in vivo* effects of EGCg on CF Sm isolates. Our results revealed important insights into the antibacterial properties of EGCg and support its future use in the prevention of biofilm formation and possible treatment of Sm biofilms in CF patients. EGCg shows promise as a novel therapeutic compound against Sm colonization and infection in patients with CF.

## Supporting Information

Figure S1 **Percentage mortality of wild-type** ***C. elegans*** **exposed during 48 h to diverse concentrations of EGCg (256, 512 and 1,024 mg/L).** Data express the mean values of two independent experiments performed in triplicated, SDs are shown.
(TIF)

Figure S2 **EGCg enhances the survival of** ***C. elegans*** **infected with** ***S. maltophilia*** **clinical isolate (Sm1).** Results are shown as mean values of three independent experiments performed in triplicated, SDs are shown.
(TIF)

File S1 **Detailed description of material and methods.**
(DOC)

## References

[1] de Vrankrijker AM, Wolfs TF, van der Ent CK (2010) Challenging and emerging pathogens in cystic fibrosis. Paediatr Respir Rev 11: 246–254. 21109184 10.1016/j.prrv.2010.07.003

[2] Hauser AR, Jain M, Bar-Meir M, McColley SA (2011) Clinical significance of microbial infection and adaptation in cystic fibrosis. Clin Microbiol Rev 24: 29–70. 21233507 10.1128/CMR.00036-10 PMC3021203

[3] Chernish RN, Aaron SD (2003) Approach to resistant gram-negative bacterial pulmonary infections in patients with cystic fibrosis. Curr Opin Pulm Med 9: 509–515. 14534404 10.1097/00063198-200311000-00011

[4] Lechtzin N, John M, Irizarry R, Merlo C, Diette GB, et al (2006) Outcomes of adults with cystic fibrosis infected with antibiotic-resistant *Pseudomonas aeruginosa* . Respiration 73: 27–33. 16113513 10.1159/000087686

[5] Gales AC, Jones RN, Forward KR, Liñares J, Sader HS, et al. (2001) Emerging importance of multidrug-resistant *Acinetobacter* species and *Stenotrophomonas maltophilia* as pathogens in seriously ill patients: geographic patterns, epidemiological features, and trends in the SENTRY Antimicrobial Surveillance Program (1997–1999). Clin Infect Dis 32(Suppl 2): ;S104–113. 10.1086/320183 11320451

[6] Mah TF, Pitts B, Pellock B, Walker GC, Stewart PS, et al (2003) A genetic basis for *Pseudomonas aeruginosa* biofilm antibiotic resistance. Nature 426: 306–310. 14628055 10.1038/nature02122

[7] Arciola CR, Campoccia D, Speziale P, Montanaro L, Costerton JW (2012) Biofilm formation in *Staphylococcus* implant infections. A review of molecular mechanisms and implications for biofilm-resistant materials. Biomaterials 33: 5967–5982. 22695065 10.1016/j.biomaterials.2012.05.031

[8] Høiby N (2002) Understanding bacterial biofilms in patients with cystic fibrosis: current and innovative approaches to potential therapies. J Cyst Fibros 1: 249–254. 15463822 10.1016/s1569-1993(02)00104-2

[9] Pompilio A, Pomponio S, Crocetta V, Gherardi G, Verginelli F, et al (2011) Phenotypic and genotypic characterization of *Stenotrophomonas maltophilia* isolates from patients with cystic fibrosis: genome diversity, biofilm formation, and virulence. BMC Microbiol 11: 159. 21729271 10.1186/1471-2180-11-159 PMC3146419

[10] McCaughey G, McKevitt M, Elborn JS, Tunney MM (2012) Antimicrobial activity of fosfomycin and tobramycin in combination against cystic fibrosis pathogens under aerobic and anaerobic conditions. J Cyst Fibros 11: 163–172. 22138067 10.1016/j.jcf.2011.11.003

[11] Waters V, Yau Y, Prasad S, Lu A, Atenafu E, et al (2011) *Stenotrophomonas maltophilia* in cystic fibrosis: serologic response on effect on lung disease. Am J Respir Crit Care Med 183: 635–640. 20889901 10.1164/rccm.201009-1392OC

[12] Waters V, Atenafu EG, Lu A, Yau Y, Tullis E, et al (2013) Chronic *Stenotrophomonas maltophilia* infection and mortality or lung transplantation in cystic fibrosis patients. J Cyst Fibros 12: 482–486. 23294530 10.1016/j.jcf.2012.12.006

[13] Brooke JS (2012) *Stenotrophomonas maltophilia*: an emerging global opportunistic pathogen. Clin Microbiol Rev 25: 2–41. 22232370 10.1128/CMR.00019-11 PMC3255966

[14] Pompilio A, Crocetta V, Confalone P, Nicoletti M, Petrucca A, et al (2010) Adhesion to and biofilm formation on IB3-1 bronchial cells by *Stenotrophomonas maltophilia* isolates from cystic fibrosis patients. BMC Microbiol 10: 102. 20374629 10.1186/1471-2180-10-102 PMC2858031

[15] Hirasawa M, Takada K (2004) Multiple effects of green tea catechin on the antifungal activity of antimycotics against *Candida albicans* . J Antimicrob Chemother 53: 225–229. 14688042 10.1093/jac/dkh046

[16] Osterburg A, Gardner J, Hyon SH, Neely A, Babcock G (2009) Highly antibiotic-resistant *Acinetobacter baumannii* clinical isolates are killed by the green tea polyphenol(-)-epigallocatechin-3-gallate (EGCG). Clin Microbiol Infect 15: 341–346. 19431221 10.1111/j.1469-0691.2009.02710.x

[17] Gordon NC, Wareham DW (2010) Antimicrobial activity of the green tea polyphenol(-)-epigallocatechin-3-gallate (EGCG) against clinical isolates of *Stenotrophomonas maltophilia* . Int J Antimicrob Agents 36: 129–131. 20472404 10.1016/j.ijantimicag.2010.03.025

[18] Xu X, Zhou XD, Wu CD (2011) The tea catechin epigallocatechin gallate suppresses cariogenic virulence factors of *Streptococcus mutans* . Antimicrob Agents Chemother 55: 1229–1236. 21149622 10.1128/AAC.01016-10 PMC3067078

[19] Steinmann J, Buer J, Pietschmann T, Steinmann E (2013) Anti-infective properties of epigallocatechin-3-gallate (EGCG), a component of green tea. Br J Pharmacol 168: 1059–1073. 23072320 10.1111/bph.12009 PMC3594666

[20] Yoda Y, Hu ZQ, Zhao WH, Shimamura T (2004) Different susceptibilities of *Staphylococcus* and Gram-negative rods to epigallocatechin gallate. J Infect Chemother 10: 55–58. 14991521 10.1007/s10156-003-0284-0

[21] Cui Y, Oh YJ, Lim J, Youn M, Lee I, et al (2012) AFM study of the differential inhibitory effects of the green tea polyphenol(-)-epigallocatechin-3-gallate (EGCG) against Gram-positive and Gram-negative bacteria. Food Microbiol 29: 80–87. 22029921 10.1016/j.fm.2011.08.019

[22] UK Cystic Fibrosis Trust Antibiotic Working Group (2009) Section 7.3: Respiratory infection with *Stenotrophomonas maltophilia*. In: Antibiotic treatment for cystic fibrosis, ed. UK: Cystic Fibrosis Trust, Bromley, Kent, UK.

[23] Clinical and Laboratory Standards Institute (2009) Methods for dilution antimicrobial susceptibility tests for bacteria that grow aerobically – Twenty second information supplement: Approved standard M07-A8. CLSI, Wayne, PA, USA.

[24] Rayamajhi M, Redente EF, Condon TV, Gonzalez-Juarrero, Riches DW, et al (2011) Non-surgical intratracheal instillation of mice with analysis of lungs and lung draining lymph nodes by flow cytometry. J Vis Exp 51: 2702. 10.3791/2702 PMC3280633 21587154

[25] Lloyd MH, Wolfensohn SE (1999) Practical use of distress scoring systems in the application of humane endpoints, p. 48–53. *In* C.F.M. Hendriksen and D.B. Morton 8ed.), Humane Endpoints in Animal Experiments for Biomedical Research. Royal Society of Medicine Press, London,UK.

[26] Stepanović S, Vuković D, Hola V, Di Bonaventura G, Djukić S, et al (2007) Quantification of biofilm in microtiter plates: overview of testing conditions and practical recommendations for assessment of biofilm production by staphylococci. APMIS 115: 891–899. 17696944 10.1111/j.1600-0463.2007.apm_630.x

[27] Pompilio A, Scocchi M, Pomponio S, Guida F, Di Primio A, et al (2011) Antibacterial and anti-biofilm effects of cathelicidin peptides against pathogens isolated from cystic fibrosis patients. Peptides 32: 1807–1814. 21849157 10.1016/j.peptides.2011.08.002

[28] Müsken M, Di Fiore S, Römling U, Häussler S (2010) A 96-well-plate-based optical method for the quantitative and qualitative evaluation of *Pseudomonas aeruginosa* biofilm formation and its application to susceptibility testing. Nat Protoc 5: 1460–1469. 20671729 10.1038/nprot.2010.110

[29] Herrmann G, Yang L, Wu H, Song Z, Wang H, et al (2010) Colistin-tobramycin combinations are superior to monotherapy concerning the killing of biofilm *Pseudomonas aeruginosa* . J Infect Dis 202: 1585–1592. 20942647 10.1086/656788

[30] Costerton JW, Stewart PS, Greenberg EP (1999) Bacterial biofilms: a common cause of persistent infections. Science 284: 1318–1322. 10334980 10.1126/science.284.5418.1318

[31] Di Bonaventura G, Stepanović S, Picciani C, Pompilio A, Piccolomini R (2007) Effect of environmental factors on biofilm formation by clinical *Stenotrophomonas maltophilia* isolates. Folia Microbiol 52: 86–90. 17571802 10.1007/BF02932144

[32] Di Bonaventura G, Spedicato I, D'Antonio D, Robuffo I, Piccolomini R (2004) Biofilm formation by *Stenotrophomonas maltophilia*: modulation by quinolones, trimethoprim-sulfamethoxazole, and ceftazidime. Antimicrob Agents Chemother 48: 151–160. 14693533 10.1128/AAC.48.1.151-160.2004 PMC310151

[33] Pompilio A, Catavitello C, Picciani C, Confalone P, Piccolomini R, et al (2010) Subinhibitory concentrations of moxifloxacin decrease adhesion and biofilm formation of *Stenotrophomonas maltophilia* from cystic fibrosis. J Med Microbiol 59: 76–81. 19762476 10.1099/jmm.0.011981-0

[34] Benelli R, Venè R, Bisacchi D, Garbisa S, Albini A (2002) Anti-invasive effects of green tea polyphenol epigallocatechin-3-gallate (EGCG), a natural inhibitor of metallo and serine proteases. Biol Chem 383: 101–105. 11928805 10.1515/BC.2002.010

[35] Zaveri NT (2006) Green tea and its polyphenolic catechins: medicinal uses in cancer and noncancer applications. Life Sci 78: 2073–2080. 16445946 10.1016/j.lfs.2005.12.006

[36] Sudano Roccaro A, Blanco AR, Giuliano F, Rusciano D, Enea F (2004) Epigallocatechin-gallate enhances the activity of tetracycline in staphylococci by inhibiting its efflux from bacterial cells. Antimicrob Agents Chemother 48: 1968–1973. 15155186 10.1128/AAC.48.6.1968-1973.2004 PMC415601

[37] Evensen NA, Braun PC (2009) The effects of tea polyphenols on *Candida albicans*: inhibition of biofilm formation and proteasome inactivation. Can J Microbiol 55: 1033–1039. 19898545 10.1139/w09-058

[38] Isbrucker RA, Bausch J, Edwards JA, Wolz E (2006) Safety studies on epigallocatechin gallate (EGCG) preparations. Part 1: genotoxicity. Food Chem Toxicol 44: 626–635. 16364532 10.1016/j.fct.2005.07.005

[39] Isbrucker RA, Edwards JA, Wolz E, Davidovich A, Bausch J (2006) Safety studies on epigallocatechin gallate (EGCG) preparations. Part 2: dermal, acute and short-term toxicity studies. Food Chem Toxicol 44: 636–650. 16387402 10.1016/j.fct.2005.11.003

[40] Wu F, Sun H, Kluz T, Clancy HA, Kiok K, et al (2012) Epigallocatechin-3-gallate (EGCG) protects against chromate-induced toxicity *in vitro* . Toxicol Appl Pharmacol 258: 166–175. 22079256 10.1016/j.taap.2011.10.018 PMC3259276

[41] O'Connell OJ, O'Farrell C, Harrison MJ, Eustace JA, Henry MT, et al (2011) Nebulized hypertonic saline via positive expiratory pressure versus via jet nebulizer in patients with severe cystic fibrosis. Respir Care 56: 771–775. 21333079 10.4187/respcare.00866

[42] Yamada H, Tateishi M, Harada K, Ohashi T, Shimizu T, et al (2006) A randomized clinical study of tea catechin inhalation effects on methicillin-resistant *Staphylococcus aureus* in disabled elderly patients. J Am Med Dir Assoc 7: 79–83. 16461248 10.1016/j.jamda.2005.06.002

[43] Navarro-Martínez MD, Navarro-Perán E, Cabezas-Herrera J, Ruiz-Gómez J, García-Cánovas F, et al (2005) Antifolate activity of epigallocatechin gallate against *Stenotrophomonas maltophilia* . Antimicrob Agents Chemother 49: 2914–2920. 15980368 10.1128/AAC.49.7.2914-2920.2005 PMC1168674

[44] Arakawa H, Maeda M, Okubo S, Shimamura T (2004) Role of hydrogen peroxide in bactericidal action of catechin. Biol Pharm Bull 27: 277–281. 14993788 10.1248/bpb.27.277

[45] Bjarnsholt T, Alhede M, Alhede M, Eickhardt-Sørensen SR, Moser C, et al (2013) The *in vivo* biofilm. Trends Microbiol 21: 466–474. 23827084 10.1016/j.tim.2013.06.002

