# Supplementary figures and images for: Effects of Green Tea Compound Epigallocatechin-3-Gallate against Stenotrophomonas maltophilia Infection and Biofilm
Source: PLoS One. 2014 Apr 1;9(4):e92876. doi: 10.1371/journal.pone.0092876 (PMC3972220; doi:10.1371/journal.pone.0092876)

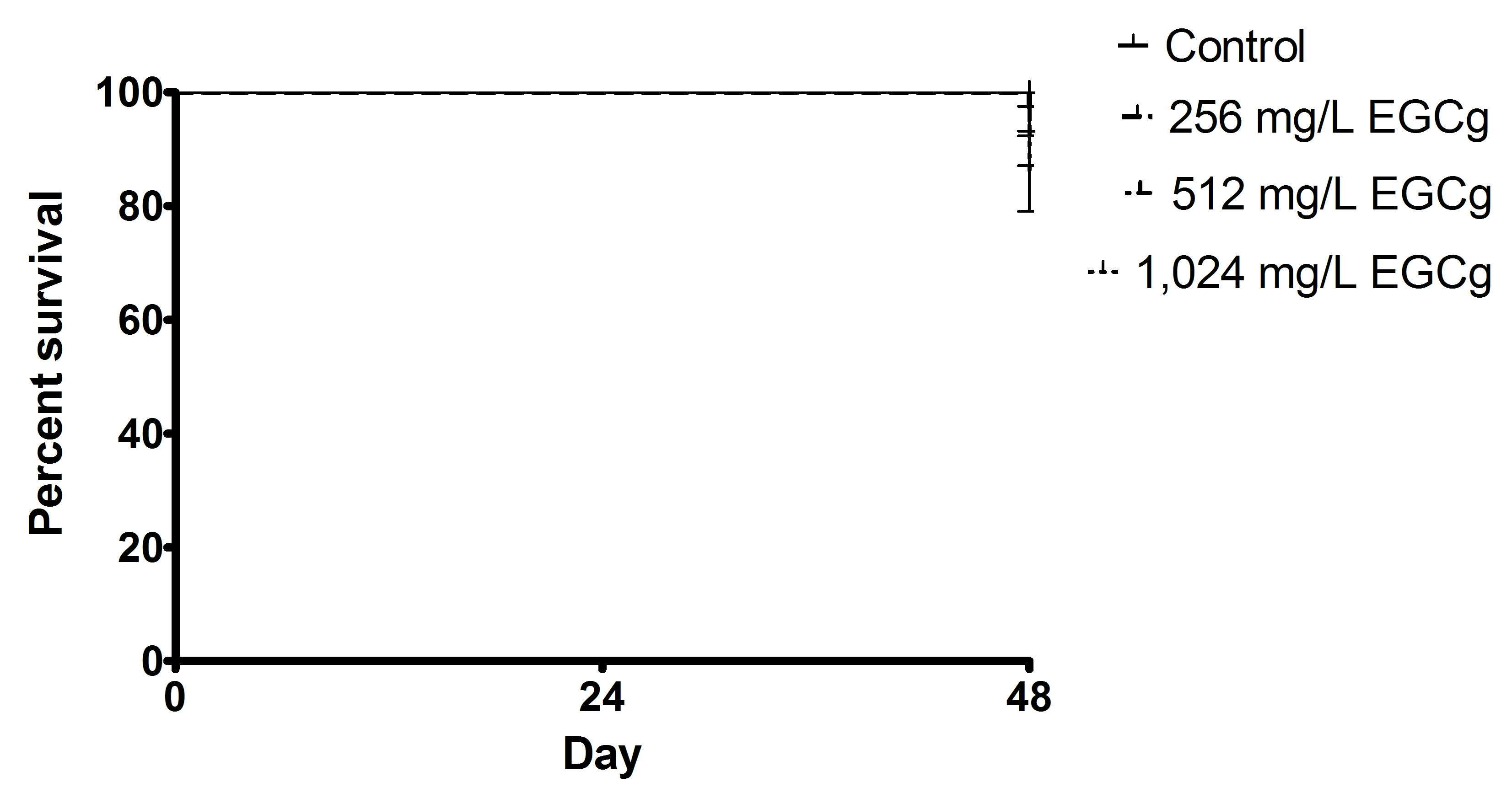

Supplement: Figure S1 — Percentage mortality of wild-type C. elegans exposed during 48 h to diverse concentrations of EGCg (256, 512 and 1,024 mg/L). Data express the mean values of two independent experiments performed in triplicated, SDs are shown. (TIF) [file pone.0092876.s001.tif]

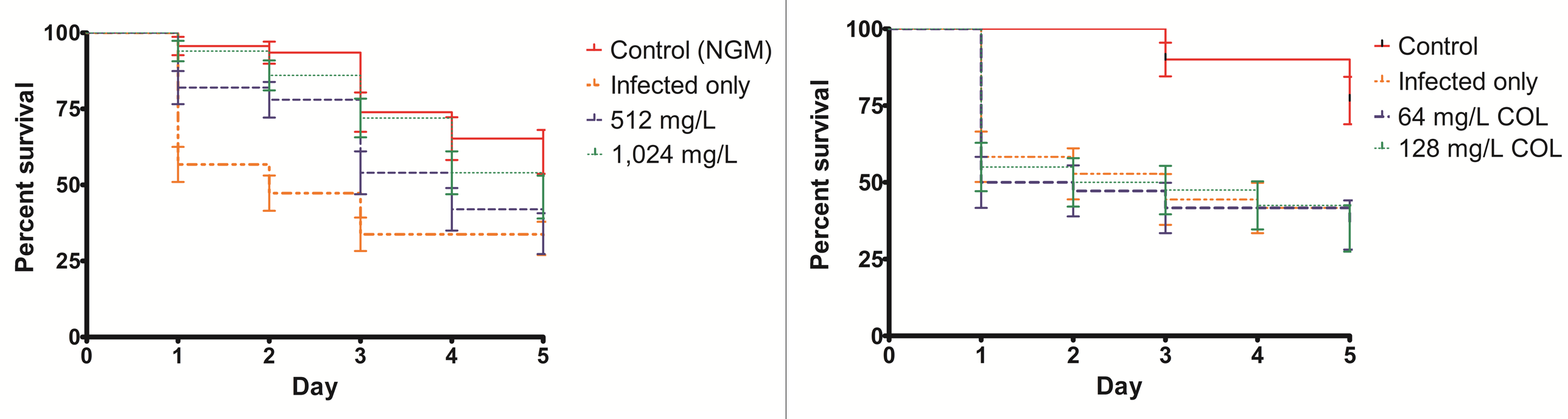

Supplement: Figure S2 — EGCg enhances the survival of C. elegans infected with S. maltophilia clinical isolate (Sm1). Results are shown as mean values of three independent experiments performed in triplicated, SDs are shown. (TIF) [file pone.0092876.s002.tif]
