# Supplementary material for: Effects of Green Tea Compound Epigallocatechin-3-Gallate against Stenotrophomonas maltophilia Infection and Biofilm
Source: PLoS One. 2014 Apr 1;9(4):e92876. doi: 10.1371/journal.pone.0092876 (PMC3972220; doi:10.1371/journal.pone.0092876)
Supplement: File S1 — Detailed description of material and methods. (DOC) [file pone.0092876.s003.doc]

**File S1 - Detailed description of material and methods.**

*Bacterial susceptibility testing of EGCg*

Susceptibility testing was carried out according to the guidelines of the Clinical and Laboratory Standards Institute (CLSI) [23]. For the broth microtitre dilution tests, 96-well microtiter plates with 50 µL of 0.5 to 1,024 mg/L EGCg per well were inoculated with 50 µL of MHB containing a bacterial cell suspension of 1×10^5^ to 5×10^5^ colony-forming units (CFUs) per mL of *S. maltophilia*. The plates were incubated for 24 h at 36 °C. Minimal inhibitory concentration (MIC) was determined as the lowest concentration able to inhibit visible growth. Minimum bactericidal concentration (MBC) was determined by transferring 10 µL aliquots of the cell suspensions that exhibited no signs of growth on broth microtitre dilution plates onto Columbia agar with 5 % (v/v) sheep blood (Oxoid). The inoculated plates were further incubated for 24 h at 36 °C. After incubation, MBC was defined as the lowest concentration that showed no growth.

*Inhalation effects of EGCg in vivo pulmonary infection*

Two hours before infection, C57BL/6 mice were nebulized for 5min with 1mL of 1,024 mg/L EGCg, 128 mg/L COL, and 1 mL 1×PBS (controls) using an inhalation device (Pari Boy® SX, Starnberg, Germany). PARI mouthpiece was adjusted for mouse inhalation by sealing the outside with parafilm and leaving only a small orifice to fit the mouse´s nose. Infection was performed with clinical isolate Sm1. Bacteria from frozen stock culture were grown on LB agar plates overnight and a suspension of bacteria was adjusted to an OD of 0.225 and consequently it was grown at 36°C for 60min with gentle agitation (125 rpm) to reach early logarithmic growth phase. Bacteria were washed with RPMI 1640 supplemented with 25mM HEPES (pH 7.4). After definition of the OD, bacteria were resuspended at a density of 1 × 10 ^7^ CFU/ 20 µL in RPMI 1640 (HEPES 25mM, pH 7.4). Mice were anesthetized with solution of xylazine (10 mg/Kg) and ketamine (50 mg/Kg) prepared in sterile 1×PBS, at final concentrations of 2 g/L and 10 g/L, respectively. Intratracheal instillation was conducted as previously described [24]. One hour post-infection, mice inhaled EGCg, COL and PBS again under the same conditions prior detailed. Clinical aspects of mice were assessed and scored as described previously [25]. Four hours after the infection, mice were euthanized by cervical dislocation. The lungs were mechanically homogenized, lysed for 10 min in 5 g/L saponin with gentle shaking (125 rpm) at 36 °C to release intracellular bacteria and washed once in RPMI 1640 (HEPES 25mM, pH 7.4). Finally, dilutions of the homogenate lung tissues were cultured on LB agar plates in triplicates. Bacterial load from mouse lungs was determined after 18 h of growth at 36 °C. In *Cftr* mutant mice, both nebulizations (2 h prior infection and 1 h post-infection) were performed with 1,024 mg/L EGCg and sterile distilled water (control), and infection was performed with 1 × 10 ^6^ CFU/ 20 µL.

*Biofilm formation assay*

The biofilm assay was performed as previously described [26] with the following modifications. Overnight cultures of *S. maltophilia* in 5 mL tryptic soy broth (TSB) (Oxoid) with an optical density 620 (OD_620_) equivalent to 1 (approximately 1×10^9^ CFU/mL) were diluted to 1:10, and 100 µL of the diluted inoculum (approximately 1×10^7^ CFU/mL) was transferred to the wells of a sterile flat-bottomed 96-well polystyrene microtiter plate (BRANDplates, BrandTech Scientific, Essex, CT, USA) and incubated for 24 h at 36 °C. Nonadherent cells were removed by washing two times with 200 µL sterile distilled water. The amount of biofilm biomass was assessed by crystal violet staining. Biofilms were stained with 125 µL of 1 % (w/v) crystal violet for 20 min. The dye solution was discarded, and the plate was washed three times with sterile distilled water and allowed to dry for 24 h at room temperature. Stained biofilms were exposed to 30 % (v/v) acetic acid for 30 min, and the OD_620_ of the extracted dye was subsequently measured.

*Effect of EGCg on biofilms*

To evaluate the disruptive effects of EGCg, we cultivated *S. maltophilia* biofilms in a 96-well microtiter plate assay as previously described [27]. Briefly, 24-h-old biofilms were treated with 100 µL of EGCg at 0.25×MIC, 0.5×MIC, or 1×MIC (Table 1) and were incubated at 36 °C for an additional 24 h. After incubation, the culture medium (TSB with EGCg or COL) was removed, and the treated biofilms were washed two times with sterile distilled water. In parallel, 7-day-old biofilms were cultivated. These biofilms were washed with sterile distilled water every 24 h during the incubation-period, and the medium was supplemented with new TSB. After the maturation period, 7-day-old biofilms were treated as stated above. To determine the metabolic activity of the biofilms, we added 100 µL aliquots of saline solution XTT (100 mg/L) and menadione (10 mg/L). The plate was incubated in the dark for 2 h at 36°C, and the absorbance was measured at 492 nm. The *in vitro* effect of EGCg on the viability of *S. maltophilia* biofilm was plotted as the ratio of viability (cells with active metabolism) in treated samples to viability in untreated samples. Control wells contained TSB without EGCg.

*Cytotoxicity of EGCg on C. elegans*

All experiments were carried out using age-synchronised young adult animals. Synchronization was performed by bleaching gravid adults with lysis buffer. Eggs were plated onto lawns of *Escherichia coli* OP50 (food source) on NGM agar media, and allowed to hatch and develop into young adults at 25 °C (approximately 42 h after synchronisation). Synchronous population was collected and washed five times using M9 solution. Final concentrations of EGCg (256 mg/L; 512 mg/L; 1,024 mg/L and 2,048 mg/L) were added to the wells, as indicated. Control consisted of animals exposed only to NGM liquid. The nematodes were incubated for 48 h at 25 °C. Data is expressed as the average of two independent experiments performed in triplicate.

*Antimicrobial effects of EGCg on S. maltophilia during infection in C. elegans*

A *C. elegans* synchronised population was obtained as described above. To infect the nematodes, 20 mL of LB broth were inoculated with a single colony of *S. maltophilia* clinical isolate (Sm1) which was then incubated overnight in an orbital incubator 36 °C with agitation (150 rpm). Aliquots of 1 mL of inoculum adjusted to 0.5 × 10^7^ CFUs/mL were added to each well containing varying concentrations of EGCg (2×MIC= 512 g/L and 4×MIC= 1,024 g/L) diluted in NGM liquid. Control wells consisted of *C. elegans* containing 1 mL NGM and 1 mL of inoculum. A control set containing only NGM medium liquid and *C. elegans* was also used. The assays were carried out in duplicate of three independent experiments with approximately 20 worms per group. The experiments were assessed every 24 h for worm mortality on continuous exposure to pathogen and EGCg under the conditions described above. Worms were considered dead if they did not move or did not exhibit muscle tone. *C. elegans* survival was plotted using Kaplan-Meier survival curves and analysed by the log rank test using GraphPad Prism software. Curves resulting in *P* values < 0.05 relative to control were considered significantly different.
